# Accessibility to cochlear implants in Belgium: State of the art on selection, reimbursement, habilitation, and outcomes in children and adults

**DOI:** 10.1179/1467010013Z.00000000078

**Published:** 2013-03

**Authors:** Leo De Raeve, Annelies Wouters

**Affiliations:** 1 Independent Information Center on Cochlear Implants (ONICI), Zonhoven, Belgium; and KIDS-Royal Institute for the Deaf, Hasselt, Belgium; 2 National Institute for Health and Disability Insurance (NIHDI), Belgium

**Keywords:** Cochlear implantation, Accessibility, Reimbursement, Rehabilitation, Outcomes

## Abstract

Belgium, and especially the northern region called Flanders, has been a center of expertise in cochlear implants and early hearing screening for many years. Some of their surgeons and engineers were pioneers in the development of cochlear implants and in 1998 Flanders was the first region in Europe to implement a universal hearing screening program for all neonates. The Belgian National Institute for Health and Disability Insurance has reimbursed cochlear implants in children and adults since 1994 and bilateral implantation in children under the age of 12 years since February 2010. These deaf children, screened and implanted early, achieve higher auditory, speech and language outcomes and increasing numbers are going to regular schools using fewer interpreters. In 2010, 93% of severe-to-profound deaf preschool children in Flanders had received cochlear implants and 25% had bilateral implants. Although on average twice as many adults as children are implanted a year in Belgium, we have less research data available from this adult population. Also very little is published about the growth curves and minimal rehabilitation requirements (intensity, duration etc.) after implantation for both children and adults. So, there still remain many challenges for the future.

## Introduction

Belgium has been a center of expertise for neonatal hearing screening and cochlear implants (CIs) for many years. During the period when William House (USA) and Graeme Clark (Australia) were developing their cochlear implants, Offeciers *et al*. (1985) from Antwerp (Belgium) were developing their Laura device. They then started to implant adults and in 1991 the first child received their cochlear implant. In 2002, the Australian company ‘Cochlear’ took over the Laura system.

The Belgian National Institute for Health and Disability Insurance (NIHDI) has reimbursed cochlear implants in children and adults from October 1994 onwards. Besides universal health care, Belgium offers also many services to people with disabilities with the aim of improving their participation, integration, and equality of opportunity in all areas of social and educational life. These services are assigned to different authorities with competencies in specific and well-defined areas. In this publication, we will mainly focus on the services and data available in Flanders, the Dutch-speaking part of Belgium.

In 1998, as the first region in Europe and 2 years before the recommendations of the Joint Committee on Infant Hearing (JCIH, 2000) were published, the Flemish public child care organization ‘Kind en Gezin’ (Child and Family) started a Universal Neonatal Hearing Screening Programme (UNHSP) in Flanders (Verhaert *et al*., 2008). Approximately 99% of all neonates are screened every year. By integrating screening, diagnosis, early intervention, and rehabilitation in one program (via a well-defined cooperation protocol between different caregivers and health services), it became a unique project (Van Kerschaver *et al*., 2007).

Evidence exists that children who receive a CI at a younger age perform better on a range of language and academic measures than children who are implanted at an older age (Anderson *et al*., 2004; Kirk *et al*., 2000; Sharma *et al*., 2002; Svirsky *et al*., 2004). There is also a growing body of research indicating that children implanted under 24 months can match the progress of normal hearing peers in some areas of language development (Geers, 2006; Hehar *et al*., 2002; Nicholas and Geers, 2006) and that many enter mainstream schooling in early primary grades (Francis *et al*., 1999; Geers, 2003; De Raeve and Lichtert, 2012).

Traditionally, a single implant was provided. However, in recent years increasing numbers of patients have received bilateral cochlear implants (Litovsky *et al*., 2010; Sparreboom *et al*., 2010). The main findings of recent research have been the benefits given by bilateral implantation for localization of sound (Greco *et al*., 2008; Beijen *et al*., 2007; Van Deun *et al*., 2009) and speech discrimination in noise (Kuhn-Inacker *et al*., 2004; Dunn *et al*., 2008; Litovsky *et al*., 2006).

## Belgian State structure and assignments of competencies for disabled persons

Belgium is a federal state, made up of three communities (the Flemish-speaking Community, the French-speaking Community, and the German-speaking Community) and three regions (the Flanders Region, the Brussels Capital Region, and the Walloon Region). The Federal government is responsible for everything which falls within the sphere of interest of all Belgians, irrespective of language, cultural, or territorial considerations. These include foreign affairs, defence, justice, finance, social security (unemployment, pensions, child benefit, health insurance) and substantial parts of public health and domestic affairs. The communities are responsible for people-related matters, such as language, culture, education, health policy (preventive medicine) and assistance to individuals (protection of youth, social welfare, support to families, …). The regions are in turn responsible for territorial matters, such as town and country planning, environment, and employment. This division of responsibilities has had far-reaching consequences in the area of care for the disabled persons, who normally need to address several authorities for (financial) assistance, support, and guidance. So the UNHSP was really a unique integrated project, taken into account the complex state structure.

## Reimbursement by the NIHDI

### Cochlear implants

Current FDA guidelines for the cochlear device recommend cochlear implantation in persons age 2 years and older with severe-to-profound deafness (i.e. pure tone average thresholds of 70 dB HL or greater), and in children 12–23 months of age with profound deafness (i.e. pure tone average thresholds of 90 dB HL or greater) (Bradham *et al*., 2009).

In Belgium, cochlear implants have been reimbursed for children and adults since October 1994 (Belgisch Staatsblad, 1994); initially only in patients with a bilateral total sensory deafness. In March 2006 (Belgisch Staatsblad, 2006), the reimbursement criteria were refined into (1) pure tone average thresholds of 85 dB HL or greater at 500, 1000, and 2000 Hz; (2) threshold of peak V in brainstem auditory evoked potentials at 90 dB HL or higher; (3) little or no benefit from hearing aids. In post-lingually deafened persons, there has to be a phoneme score, using monosyllabic words at 70 dB, of less than 30% with hearing aids, which indicates that they do not give sufficient benefit.

In case of mental retardation, psychological, or psychiatric problems, the family situation and the rehabilitation plan must be demonstrated in a psychological report. After implantation, long-term auditory/speech therapy and follow-up are required and reimbursed, until the age of 18 years for children and two years for adults. All this should be coordinated by a specialized multidisciplinary team.

A pilot project on bilateral implantation was initiated in 2003 by the NIHDI in which 42 children under 12 years have received a contralateral CI. The children had to meet several criteria in order to be considered for this project: presence of a full insertion of the electrode array, having showed good cooperation with rehabilitation and good audiometric results with their first CI and a normal anatomy of the second ear (cochlea and cochlear nerve). The outcomes of this project (Scherf *et al*., 2009a; Scherf *et al*., 2009b; van Deun *et al*., 2009) justified a standard reimbursement for the second implant in children younger than 12 years. This has been official policy since February 2010 (Belgisch Staatsblad, 2009). The indication for a second cochlear implant has also been broadened to include children between 12 months and 18 years with an auditory neuropathy. In case of meningitis with a threatened bilateral ossification, a contralateral implant is reimbursed up to the age of 18 years. For adults, there is no formal reimbursement of a second cochlear implant, either in case of auditory neuropathy, or in case of meningitis with ossification.

**Figure 1:**
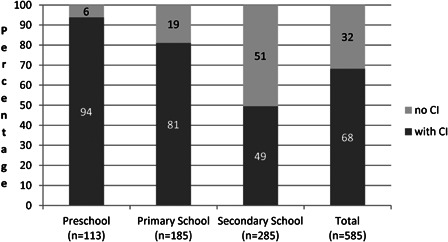
Percentage of deaf school age children in Flanders wearing a cochlear implant in February 2010.

The result of all this is that in 2010, 94% of all deaf (‘deaf’ in this publication has to be understood as an average bilateral hearing loss at 500, 1000, 2000 and 4000 Hz > 90 dB SPL unaided) children in Flanders, who were of preschool age (2.6–6.0 years) and within the criteria for cochlear implantation, were wearing a cochlear implant (Fig. 1). For those of primary school age (6.0–12.0 years) this figure was 81% and at secondary school age it was 49% (De Raeve and Lichtert, 2012). So nearly every deaf born child in Flanders now receives a CI if they are within the criteria. Such figures clearly demonstrate that in 6 years time, 80–90% of all Flemish school-aged children who are deaf will be wearing one or two CIs if within the criteria.

Concerning adults, we do not have the same detailed information that we have for children. It is difficult to estimate the percentage of eligible adults receiving a CI. Based on the numbers of the WHO (2010) and on the current selection criteria in most countries (bilateral hearing loss >85–90 dB HL) 8 to 10% of the people in developed countries have a hearing loss. Of these, 10% (or 1% of the total population) have a severe-to-profound hearing loss and could be CI candidates. Some adults with a severe-to-profound hearing loss may not consider a CI for several reasons: ossified cochleas, congenitally deaf adults who have never used hearing aids.

**Figure 2:**
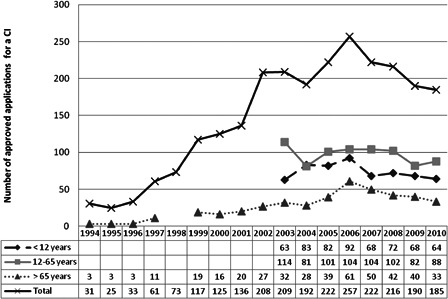
Overview of the number of approved applications for a reimbursement of a CI in Belgium from 1994 till 2010.

Fig. 2 gives an overview since 1994 of the number of applications submitted for reimbursement of a cochlear implant and for which there was an approval from the NIHDI. We only have detailed information since 2005, but it is still clear that there has been a continuous increase till 2006. Since then there is a small decrease for nearly all the age categories and we cannot explain why this happened.

**Figure 3:**
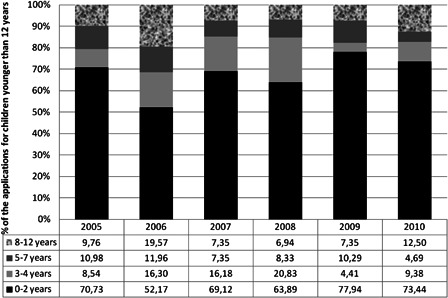
The percentage of applications for a reimbursement of a CI depending on the age of the child as function of the total number of applications for children younger than 12 years.

Looking at the number of applications submitted for reimbursement of a CI for all children under the age of 12 years during the period 2005–2010, it can be seen in Fig. 3 that between 2005 and 2010, on average 68% of patients were younger than 2 years, 13% were children of age 3–4 years, 9% were under the age of 5–7 years, and 11% were children between 8 and 12 years old.

## (Bilateral) Cochlear implants in children

### Selection of pediatric CI candidates

Initially, mainly children who were totally deaf, with normal intelligence and a normal cochlea received a CI. But over time these criteria have become less stringent and we are now implanting children at younger ages, with greater amount of residual hearing, additional needs, and even malformed cochleas.

Since the implementation of the UNHSP in Flanders, the average age of fitting a hearing aid has been reduced to 4 months and referral for a cochlear implant assessment is usually accomplished before the age of nine months (Philips *et al*., 2009). When appropriate, children now receive a CI by their first birthday and since 2004 the average age of implantation is between 14 and 16 months. Looking at the preschool population (age 2;6–6;0 years) of bilaterally deaf children in 2010, 94% of all these children in Flanders have received a cochlear implant and of this group 25% are wearing bilateral implants (De Raeve and Lichtert, 2012). The number of bilaterally implanted children is expected to increase quickly, because bilateral implants have been reimbursed in Belgium in children under the age of 12 years since February 2010 (Belgisch Staatsblad, 2009).

### Support and rehab

As mentioned before, early intervention is integrated in the UNHSP in Flanders. This means that immediately after detecting the baby's hearing loss, families are referred to a ‘service’ center (specialized ENT-department, early intervention team and/or rehab center) which organizes further support, rehabilitation, and follow-up of the child and family. These centers provide the families with a lot of information concerning hearing tests, hearing devices, and communication. During home visits or conversations at the center, there is emphasis on helping the family to cope with this new and mostly unexpected situation and gradually providing them with a lot of new information on the effects of having a deaf or hard of hearing child (De Raeve, 2006).

Some service centers in Belgium, most of them in cooperation with a rehabilitation center, offer daycare for infants and toddlers with a hearing loss, often together with hearing children. Depending on the childrens' individual needs, speech and language therapists, audiologists, psychologists, physiotherapists, and care staff of the daycare or rehab center can stimulate the children's auditory, speech, language, motor, social emotional, or cognitive skills and can provide instruction to parents in small groups or on an individual basis. In general, children visit this specialized (rehab) center two to three times a week. For children, a multidisciplinary approach is reimbursed until the age of 18 years. The alternative, monodisciplinary speech therapy, is reimbursed for a period of 2 years (which can be prolonged if necessary). Unfortunately, there are no official published figures available about the actual minimal demand and consumption of rehabilitation sessions or on the intervening discipline.

**Figure 4:**
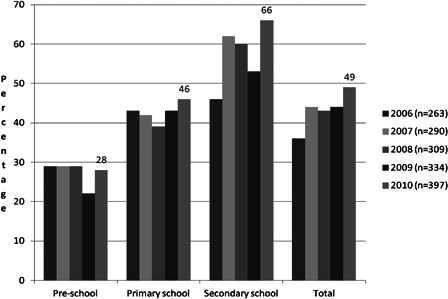
Percentage of Flemish deaf children with a cochlear implant in going to regular schools at pre-, primary and secondary school level from 2006 till 2010.

At the age of 2.6 years, children in Belgium can start going to a preschool. As can be seen in Fig. 4 nearly 75% of implanted children start in a special school for the deaf. Possible reasons could be: spoken language delay, preference for smaller classes (average number is 6 in a special school for the deaf and 20 in mainstream), more opportunities to receive regular (in most cases daily) speech and language therapy, good classroom acoustics, etc. When parents decide to change to a mainstreamed setting, children with a profound hearing loss (>90 dB HL at both ears) can receive 4 hours of support per week from a teacher of the deaf or speech/language therapist from the nearest school for the deaf. It is also important to know that we do not have a specialized training for ‘teachers of the deaf’ in Belgium. There is only generic training for teachers working with children with all kinds of special needs (Lichtert, 2010). All students with a hearing loss also have the opportunity to access resources like FM-devices; at secondary level, deaf students can ask for an additional sign language interpreter or note taker.

### Outcomes

Looking at deaf school-age children with cochlear implants in Flanders, it can be seen in Fig. 4 that the percentage of deaf children in mainstream schools wearing a cochlear implant is gradually increasing from preschool through primary school and secondary school.

In 2010 two out of three of the students at secondary level with a cochlear implant were attending regular schools. This is an increase of 40% compared with a similar study carried out in the same region in 1999 (De Raeve, 2001; De Raeve, 2006).

**Figure 5:**
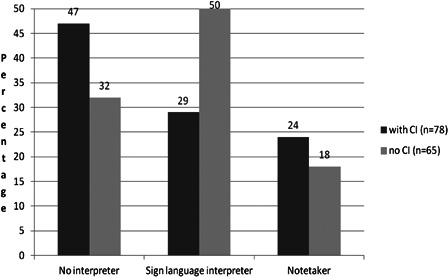
General comparison of the use of an interpreter by deaf children with and without CI at secondary level during school year 2009–2010.

Looking at the general data of deaf pupils, with and without CI, it can be seen in Fig. 5 that 53% of the deaf students wearing a cochlear implant use an interpreter (sign language interpreter or note taker) at secondary level, comparing to 68% in the group without CI. Additionally, fewer students with a CI use a sign language interpreter, but more prefer a note taker compared with deaf students without a CI.

So it can be expected that in the near future, fewer deaf students with cochlear implants will use interpreters. In difficult listening situations some of them will probably prefer extra support from a note taker (or speech to text software) instead of a sign language interpreter.

## (Bilateral) Cochlear implants in adults

### Selection CI candidates

Current guidelines for deaf adults in Belgium permit implantation in case of an average bilateral hearing loss above 85 dB HL on 500, 1000, and 2000 Hz. In post-lingually deafened adults, a phoneme score, using monosyllabic words at 70 dB, of less than 30% has to be recorded with hearing aids which indicates that they do not give sufficient benefit. As mentioned earlier, bilateral implants are not reimbursed for adults.

### Support and rehab

Concerning support and rehab for adults who have received a CI, there is a huge difference compared with children. Based on the Belgian health care system, adult CI users can receive a maximum of two years multidisciplinary therapy after implantation. Monodisciplinary therapy (only speech/auditory therapy) is an alternative which is also reimbursed for a period of 2 years and it appeals more to adults than to children.

Under the compulsory health insurance, it is required to have long-term speech/auditory follow-up after implantation. But, in practice it seems, from feedback, that some adults receive no or limited therapy after implantation. However, some of them appear still very successful CI users. Regrettably, there are no published data available regarding the actual demand and consumption of rehabilitation sessions after CI implantation in adults.

### Outcomes

Compared with children, there is less follow-up and very little available research data on the general population of adult CI users. Just a few CI clinics have published on the outcomes from their population. Vermeire *et al*. (2005) from the University Hospital Antwerp conducted a study on 89 adult CI users of which 25 were older than age 70 years. They came to the conclusion that although the audiological performances of the elderly group were significantly lower than those of the younger age groups, the quality-of-life outcomes of the geriatric group were similar to younger adult cochlear implant recipients.

## Discussion

Belgium has a very high number of early implanted children. The reason of this can be found in the fact that Belgium was one of the pioneers of cochlear implantation and the Dutch speaking part of Belgium (Flanders) was the first region in Europe to implement a UNHSP. The government health care system supports cochlear implants in adults and in children from the early years and they have been reimbursed in adults and children since 1994. Bilateral implants in children have been reimbursed since February 2010.

Children with a hearing loss are mostly supported on a multidisciplinary way by early intervention teams, rehab centers and/or CI-teams and in some areas there are specialized daycare centers available for children with a hearing loss. Although in 2010 most deaf children wearing cochlear implants started in a special preschool for the deaf, by primary school age 45% were going to a regular mainstream school and this increased to 67% at secondary level.

There is also a growing body of research done on this Belgian population indicating that the auditory, speech, language, and academic outcomes of these children who were screened and implanted early is better than ever before. This results in more deaf children going to regular schools (Schauwers *et al*., 2004, 2005, 2008; Scherf *et al*., 2007, 2009a, 2009b; van Deun *et al*., 2009a, 2009b; Philips *et al*., 2009; van der Kant *et al*., 2010; Tait *et al*., 2007, 2010; De Raeve, 2010; Baudonck *et al*., 2010).

Knowing that reimbursement of bilateral implantation is possible in Belgium for deaf children since 2010, we are convinced that the number of mainstreamed children and also their school performances will increase further in the near future. Because of this changing population of children with a profound-severe hearing loss, their needs are also changing, which means that the staff supporting all these children also have to be updated. All these represent a major challenge for the caregivers and educational services. They have to adapt their way of working and they must ensure that their staff have the skills to meet the challenges. They will need to be flexible, continually updated with the technology and changing expectations and to receive ongoing professional training, in order to provide an environment which will utilise the useful hearing while meeting the linguistic and curricular needs of the children, and to meet the psycho-social needs of this group as they grow through adolescence, and to work with other professionals. The competent authorities face challenges in the field of changing needs which require continuously evolving policies.

Although twice more adults than children are implanted every year in Belgium, we have less research data available from this adult population. Although deaf adults who receive a CI must receive speech/auditory follow-up after implantation (with a maximum of 2 years reimbursed), in practice it seems not all adults make use of these services. So there may be a need to explore the current actual care for adult CI users in Belgium with examination of possible pitfalls and solutions. We also know that a cochlear implant improves the quality of life of these deaf adults, even the elderly population.

Currently, there are no available clinical guidelines with details of support and therapy needs for adults receiving a CI, nor for children. So, there still remain many challenges for the future.
